# Clinical characteristics and a 2-year follow-up of unsatisfactory conventional Pap smears: a retrospective case–control study

**DOI:** 10.1038/s41598-022-19784-3

**Published:** 2022-09-13

**Authors:** Chin-Tzu Tien, Pei-Chen Li, Chi-Jui Chen, Dah-Ching Ding

**Affiliations:** 1 Department of Obstetrics and Gynecology, Hualien Tzu Chi Hospital, Buddhist Tzu Chi Medical Foundation, Tzu Chi University, No. 707, Chung-Yang Rd., Sec. 3, Hualien, 970 Taiwan, ROC; 2 Department of Internal Medicine, Hualien Tzu Chi Hospital, Buddhist Tzu Chi Medical Foundation, Tzu Chi University, Hualien, 970 Taiwan, ROC; 3 Institute of Medical Sciences, Tzu Chi University, Hualien, 970 Taiwan, ROC

**Keywords:** Biomarkers, Diseases, Health care, Oncology, Pathogenesis

## Abstract

The objective of this study was to conduct a 2-year follow-up of individuals having unsatisfactory reports of Pap smears and to analyze the contributing factors. This was a retrospective study at a medical center that performed about 5000–6000 Pap smears annually in Eastern Taiwan. Women who had unsatisfactory results due to scant cellularity between January 1, 2015–December 31, 2016, were included in this study. The control group comprised age-matched women with normal Pap smears at a 1:4 ratio, during the same period. The clinical characteristics and the 2-year outcomes were followed. Patients who were unavailable for follow-up assessments or who had insufficient clinical information were excluded. Student’s *t*-test and chi-square test were used for continuous and categorical variables, respectively. Statistical significance was defined as a *p*-value < 0.05. A total of 887 Pap smears were included. A total of 717 and 170 women had normal Pap and unsatisfactory Pap tests, respectively. After excluding women who were unavailable for follow-up, the final analysis included 248 and 67 women with normal and unsatisfactory Pap tests, respectively. The mean age was not significantly different between the two groups (49.97 ± 10.69 and 51.61 ± 11.28 years in the unsatisfactory Pap and control groups, respectively [*p* > 0.05]). The percentage of menopause and vaginal discharge were significantly different between the two groups. Multivariate analysis revealed that premenopausal status, increased discharge were associated with the risk of unsatisfactory Pap tests. Of the 67 women with unsatisfactory Pap tests, all tested negative for any malignancies at a 2-year follow-up assessment. Women with increased vaginal discharge and without menopause were at an increased risk of having an unsatisfactory Pap test. Our results indicate that an unsatisfactory Pap smear due to scant cellularity might not increase the risk of intraepithelial neoplasia or cancer after 2 years. Further, large-scale studies with longer follow-up periods are required.

## Introduction

Cervical cancer is one of the most commonly occurring cancers in women worldwide^1^. In Africa, cervical cancer is the leading cause of cancer-related deaths; China and India together contribute more than one-third of the global burden of cervical cancer. Interestingly, the incidence decreases in high-resource countries^1^. In Taiwan, the incidence rate of cervical cancer is the 11th highest among women, and the mortality rate of cervical cancer is the 7th highest among all women cancers^2^. The mortality rate of cervical cancer has declined by utilizing Pap smear tests as a screening tool^2^. Additionally, the prevalence of cervical intraepithelial neoplasia (CIN) 3 + decreases in response to the human papillomavirus (HPV) vaccination, and new triage tests (primary HPV screening, co-testing with HPV screening and cervical cytology, and cervical cytology alone) have been implemented^3^.

For decades, the Pap smear has been a widely used tool utilized in screening for cervical cancer. However, most women report unpleasant feelings when having a Pap smear and further unsatisfactory results are additionally troublesome^4^. The 2014 Bethesda System criteria for Pap test reporting included quantitative criteria, which arbitrarily defined an “estimated minimum of approximately 8000–12,000 well-preserved and well-visualized squamous cells or squamous metaplastic cells” as adequate for evaluation with conventional smears, and “minimum of 5000 cells” as adequate for evaluation with liquid-based preparations^5^. Unsatisfactory Pap smears are often dismissed as potential patient safety issues. Previous studies found that unsatisfactory Pap smears have an increased risk of CIN2 + disease, which leads to missed detection in patients who do not complete follow-up assessments^6^.

In our hospital, most of the unsatisfactory results were due to insufficient cellularity. Therefore, the objective of this study was to increase the quality of patient care by evaluating the possible clinical factors and 2-year follow-up results of patients who had an unsatisfactory Pap smear due to scanty cells.

## Materials and methods

### Data source and study design

This is a case–control study from a medical center in Eastern Taiwan that performed 5,000–6,000 pap smears annually. The study protocol was approved by the Research Ethical Committee of Hualien Tzu Chi Hospital (IRB 108–132). We used stratified sampling to define our final study population and reviewed the Pap smear results obtained between January 1, 2015–December 31, 2016, in Hualien Tzu Chi Hospital and then at a 2-year follow-up.

### Study population

Using conventional techniques, Pap smears were performed by eight gynecologists and eleven postgraduate years (PGY) physicians. Conventional techniques were described below. Cells from the exocervix and endocervix are extracted using a brush (Fusin Corp., Taipei, Taiwan), and the brush is rotated 360° twice to extract a sufficient number of cells. Then, the brush is immediately smeared over a glass slide in a rotary manner and fixed in 95% ethyl alcohol. After fixation, the glass slide is sent and checked by cytopathologists.

During the study period, 10,887 women underwent conventional Pap smears, and 439 women had unsatisfactory results (4.03%). The extracted data only revealed the number of unsatisfactory Pap smears due to insufficient cellularity. The control group was selected by age-matching women at a 1:4 ratio, during the same period. Demographic information, menopause status, history of radiation, hysterectomy status, previous HPV vaccination, and follow-up Pap smear results were collected from our hospital’s electronic medical records database. We excluded women without adequate demographic information and those who were unavailable for follow-up after 2 years.

### Outcome measures

The primary outcome was the detection of CIN2 + , CIN3 + , and cervical cancer at the 2-year follow-up, after unsatisfactory pap smears. Evaluation of the clinical characteristics associated with unsatisfactory Pap smear results was the secondary outcome.

### Statistical analysis

Categorical data are presented as numbers (percentages). The chi-square test was used for categorical variables. Statistical analyses were performed using software (SPSS, 21.0 version; IBM Corp, Armonk, NY). Statistical significance was set at *p* < 0.05.

### Ethical approval

The study was conducted in accordance with the guidelines of the Declaration of Helsinki and approved by the Research Ethics Committee of Hualien Tzu Chi Hospital (IRB 108–132).

### Informed consent

Informed consent was waived due to a low risk involved in this study, and it was approved by the appropriate Committee.

## Results

### Demographics

**Figure 1:**
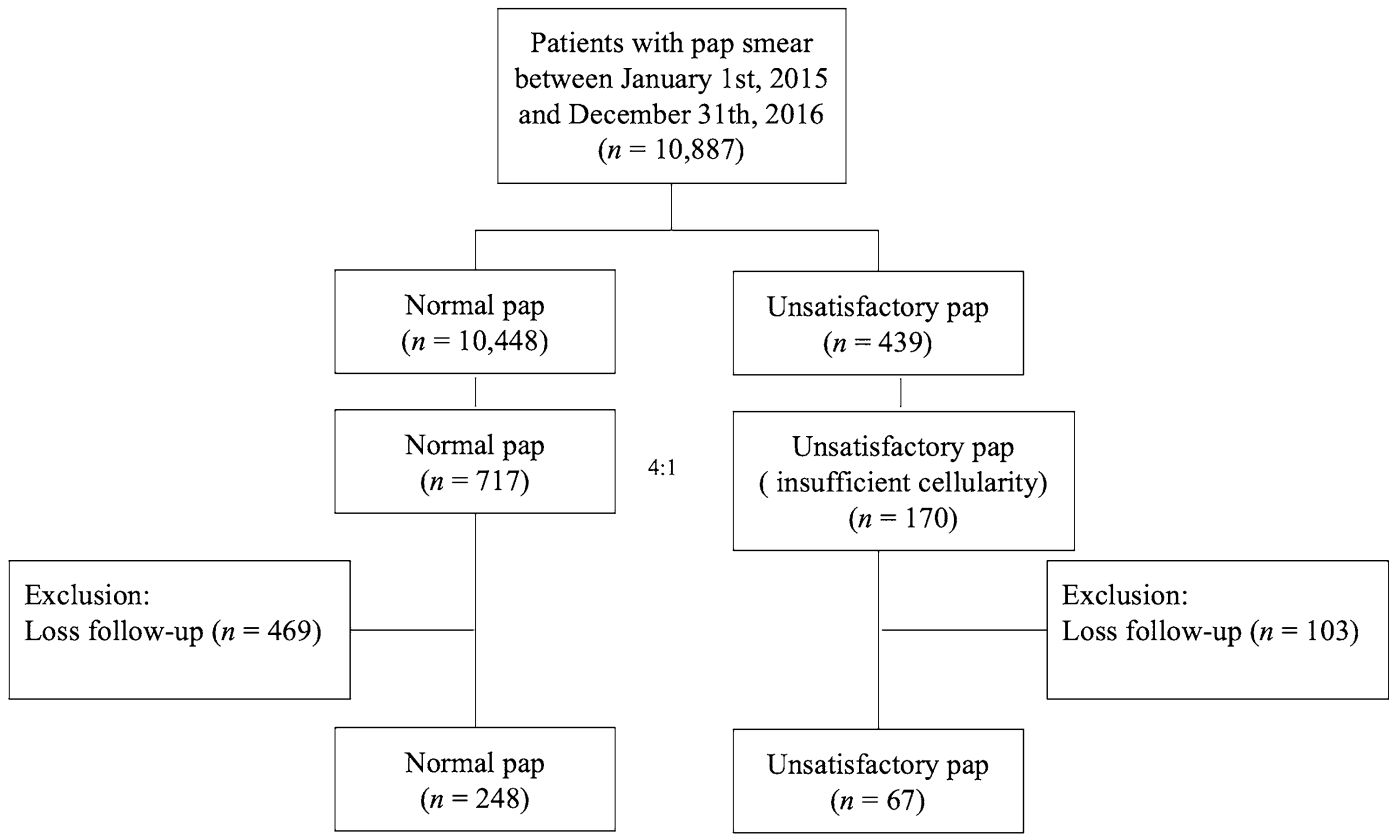
Flowchart of the study.

During the study period, 10,887 women underwent conventional Pap smear, and 439 women had unsatisfactory results (4.03%). Finally, the test results of 887 women who fulfilled the inclusion criteria for our study were considered. Among them, 717 (80.83%) and 170 (19.17%) women had normal and unsatisfactory Pap tests, respectively (Fig. 1). After excluding patients with insufficient clinical information, 248 (78.7%) and 67 (21.3%) women with normal and unsatisfactory Pap tests, respectively, were included in the final analysis (Fig. 1).

In order to know the potential risk factors of unsatisfactory Pap, we compared the clinical characteristics of the unsatisfactory Pap group with the normal Pap group. Demographic characteristics are shown in Table 1. The mean age was 49.97 ± 10.69 and 51.61 ± 11.28 years in the unsatisfactory and the control Pap group, respectively. The age distribution, percentage of menopause, and vaginal discharge were significantly different between the two groups.

**Table 1:** Patient Demographics (n = 315).

Item	Pap smear		Total	*P*-value
	Normal	Unsatisfactory		
*N*	248	67	315	
Age	51.61 ± 11.28	49.97 ± 10.69	51.26 ± 11.16	0.286
**Age Group**	–	–	–	0.062
< 40 y/o	42 (17.0%)	11 (16.4%)	53 (16.9%)	
40–49 y/o	58 (23.5%)	26 (38.8%)	84 (26.8%)	
50–59 y/o	80 (32.4%)	19 (28.4%)	99 (31.5%)	
> = 60 y/o	67 (27.1%)	11 (16.4%)	78 (24.8%)	
Menopause Age	49.55 ± 3.76	49.91 ± 4.30	49.60 ± 3.82	0.685
**Menopause Age Group**	–	–	–	0.147
< 48 y/o	35 (24.5%)	5 (22.7%)	40 (24.2%)	
48–51 y/o	59 (41.2%)	5 (22.7%)	64 (38.8%)	
> = 52 y/o	49 (34.3%)	12 (54.5%)	61 (37.0%)	
**Menopause**	–	–	–	< 0.001*
No	90 (36.3%)	41 (61.2%)	131 (41.6%)	
Yes	158 (63.7%)	26 (38.8%)	184 (58.4%)	
**Hysterectomy**	–	–	–	0.052
No	216 (87.1%)	64 (95.5%)	280 (88.9%)	
Yes	32 (12.9%)	3 (4.5%)	35 (11.1%)	
**Radiation**	–	–	–	0.381
No	247 (99.6%)	66 (98.5%)	313 (99.4%)	
Yes	1 (0.4%)	1 (1.5%)	2 (0.6%)	
**HPV vaccine**	–	–	–	0.742
No	236 (95.2%)	65 (97.0%)	301 (95.6%)	
Yes	12 (4.8%)	2 (3.0%)	14 (4.4%)	
**Discharge**	–	–	–	0.009*
No	240 (96.8%)	59 (88.1%)	299 (94.9%)	
Yes	8 (3.2%)	8 (11.9%)	16 (5.1%)	
**Who**	–	–	–	0.079
OBG	237 (95.6%)	67 (100.0%)	304 (96.5%)	
PGY	11 (4.4%)	0 (0.0%)	11 (3.5%)	

Data are presented as n or mean ± standard deviation.

*p-value < 0.05 was considered statistically significant after test.

OBG: obstetrics and gynecology; PGY: postgraduate years 1 and 2.

### Risk of unsatisfactory Pap results

In multivariate analysis (Table 2), premenopausal status and increased vaginal discharge, but not age, hysterectomy status, history of radiation exposure, or HPV vaccination status, were associated with the risk of unsatisfactory Pap results.

**Table 2:** Factors associated with unsatisfactory pap tests. (*n* = 315).

	Crude		Adjust	
	Odds Ratio (95% CI)	*p* value	Odds Ratio (95% CI)	*p* value
**Age group**	–	–	–	–
< 40 y/o	Ref		Ref	
40–49 y/o	1.71 (0.76, 3.84)	0.193	2.00 (0.85, 4.72)	0.111
50–59 y/o	0.91 (0.39, 2.08)	0.818	2.39 (0.78, 7.27)	0.126
> = 60 y/o	0.63 (0.25, 1.57)	0.320	1.87 (0.51, 6.81)	0.345
Menopause (Yes vs. No)	0.36 (0.21, 0.63)	< 0.001*	0.33 (0.13, 0.83)	0.018*
Hysterectomy (Yes vs. No)	0.32 (0.09, 1.07)	0.064	0.33 (0.07, 1.50)	0.150
Radiation (Yes vs. No)	3.74 (0.23, 60.63)	0.353	17.96 (0.75, 428.83)	0.074
HPV vaccine (Yes vs. No)	0.61 (0.13, 2.77)	0.518	0.48 (0.09, 2.56)	0.391
Discharge (Yes vs. No)	4.07 (1.47, 11.29)	0.007*	3.26 (1.10, 9.67)	0.033*
**Who**	–	–	–	–
OBG	Ref		Ref	
PGY	0.00 (NA)	0.999	0.00 (NA)	0.999

Data are presented as Odds ratio(95% CI).

*p-value < 0.05 was considered statistically significant after test.

Ref.: reference, NA: Not available.

OBG: obstetrics and gynecology; PGY: postgraduate years 1 and 2.

### Risk of unsatisfactory Pap results among menopausal women

There was an unequal distribution based on the menopausal status in the two groups; therefore, we performed a subgroup analysis of the risk of unsatisfactory pap results among menopausal women (Table 3). There was no association with age, hysterectomy status, history of radiation exposure, HPV vaccination status, or vaginal discharge except Family Medicine resident doctors who performed pap smears (Table 3).

**Table 3:** Factors associated with no unsatisfactory pap tests among menopause women. (*n* = 184).

	Crude		Adjust	
	Odds Ratio (95% CI)	p value	Odds Ratio (95% CI)	*p* value
**Menopause age group**	–	–	–	–
< 48 y/o	Ref		Ref	
48–51 y/o	0.59 (0.16, 2.19)	0.434	0.46 (0.11, 1.87)	0.275
> = 52 y/o	1.71 (0.55, 5.31)	0.350	1.34 (0.37, 4.77)	0.656
Hysterectomy (Yes vs. No)	0.51 (0.15, 1.82)	0.302	0.38 (0.07, 1.97)	0.248
Radiation (Yes vs. No)	6.28 (0.38, 103.66)	0.199	19.17 (0.78, 470.27)	0.070
HPV vaccine (Yes vs. No)	0.00 (NA)	0.999	0.00 (NA)	0.999
Discharge (Yes vs. No)	2.07 (0.21, 20.66)	0.537	2.01 (0.18, 21.88)	0.566
**Who**	–	–	–	–
OBG	Ref		Ref	
PGY	0.00 (NA)	0.999	0.00 (NA)	0.999

Data are presented as Odds ratio(95% CI).

*p-value < 0.05 was considered statistically significant after test.

OBG: obstetrics and gynecology; PGY: postgraduate years 1 and 2.

### -year follow-up results

Table 4 shows the 2-year follow-up results and notably, all the 67 women with unsatisfactory Pap results tested negative for malignancy. In the control group, of the 248 women, one woman (0.4%) was found to have CIN3 + , and the rest were negative for any malignancy. There was significant difference between the two groups regarding reacdtive changes (*p* = 0.03).

**Table 4:** Comparison of 2-year follow-up results (*n* = 315).

Item	Pap smear		*P*-value
	Normal	Unsatisfactory	
N	248	67	
**2Y-FU Result**	–	–	0.030*
Within normal limit	196 (79.0%)	46 (68.7%)	
Reactive changes	36 (14.5%)	19 (28.4%)	
Atrophy with inflammation	14 (5.6%)	1 (1.5%)	
Unsatisfactory	1 (0.4%)	1 (1.5%)	
CIN3	1 (0.4%)	0 (0.0%)	

Data are presented as n or mean ± standard deviation.

*p-value < 0.05 was considered statistically significant after test.

CIN3: cervical intraepithelial neoplasia 3.

## Discussion

Based on the Bethesda system, there are various factors that contribute to an unsatisfactory pap smear, including hemorrhagic specimens, obscuring inflammation, low cellularity, or preservation of cells^7^. In our previous study, among women with a reactive cellular change Pap test who underwent colposcopy biopsy (*n* = 49), there were 30 women with normal findings (61%), 9 with mild dysplasia (18.3%), 1 with moderate dysplasia (2%), 2 with severe dysplasia (4%), 2 with squamous cell carcinoma (4%), 1 with adenocarcinoma (2%), and 4 with benign lesions (8.1%)^8^. In this study, we further selected the unsatisfactory Pap results due to scant cellularity and compared them with the control group to evaluate the risk factors and 2-year follow-up results.

Sharma et al. performed a case–control study in a tertiary care institute in which older age groups and cervical erosion had a significant association with unsatisfactory Pap smears^4^. Paulin et al. also found that older women were at a greater risk of having unsatisfactory Pap smear results. However, earlier dates of the menstrual cycle and postpartum status were not significantly associated with unsatisfactory smears^9^. In our study, contrary to previous studies, older age was not associated with unsatisfactory Pap results. This may be because of improvements in the smear technique^10^.

Lu et al. reported that the incidence of low-cellularity in Pap tests in patients undergoing pelvic radiotherapy or chemotherapy was unacceptably high (using 8,000 cells per slide as the criteria)^11^. They suggested that 2,000 cells per slide could be used as a satisfactory threshold in this context^11^. Gupta et al. found that older age, history of hysterectomy, radiotherapy, or chemotherapy were significantly associated with unsatisfactory Pap results^12^. The main morphological determinants of unsatisfactory smears are scant cellularity and obscuring inflammation. In our study, pelvic radiotherapy or a previous hysterectomy was not significantly associated with unsatisfactory Pap results after adjustment.

Thamsborg et al. found that the proportion of unsatisfactory samples and samples with missing pathology diagnoses decreased from 6% to 1.2% after HPV vaccination. However, they concluded that the results were due to shifts from using conventional cytology to using liquid-based cytology instead of the HPV vaccination^13^. In our study, the HPV vaccination was not found to be significantly associated with an unsatisfactory Pap smear.

Sharma et al. found that white vaginal discharge and lower abdominal pain were associated with unsatisfactory Pap smears^4^. Another study including a total of 5,662 women who underwent a Pap smear test revealed that 4.5% (252/5,662) had unsatisfactory Pap smear results^14^. They found that vaginal bleeding (Odds ratio [OR] = 2.02, 95% confidence interval [CI] = 1.30–3.16, *p* = 0.002), postpartum status (OR = 1.92, 95% CI = 1.23–3.01, *p* = 0.004), and endocervical polyps (OR = 2.62, 95% CI = 1.39–4.947, *p* = 0.003) were associated with unsatisfactory Pap results^14^. In our study, a history of increased vaginal discharge was found to be significantly associated with an unsatisfactory Pap smear. Based on this finding, gynecologists/health care providers should be aware of sampling conditions such as increased discharge or other symptoms (i.e., vaginal bleeding, postpartum status, or endocervical polyps) to avoid unsatisfactory Pap results.

The most common cause of unsatisfactory results in our study was scant cellularity. Optimal collection techniques can also reduce the proportion of scant cellularity. In our previous study, a modified technique using normal saline as a lubricant and performing a smear on two glass slides effectively reduced the percentage of unsatisfactory Pap smears (from 4.71% to 0.33% in 2016 and 2018 [*p* < 0.001])^10^. Ranjana et al. found similar results that reduced the rate of unsatisfactory smears when using a liquid-based cytology technique compared to the conventional pap smear technique (6.67% to 1.67%)^15^. Doing Pap smear at 10-14th day of menstrual cycle/5 days after menstrual period is the best time to minimize the scant cellularity^4^. Measures to reduce unsatisfactory Pap results may also decrease the burden of follow-up work.

Adam et al. followed up 172 cases of unsatisfactory Pap results for 5 years and found that 25 (14.5%) had squamous abnormalities but no significant differences were found when compared with control groups^16^. Hock et al. studied 1,972 cases of unsatisfactory Pap results and followed-up for 5 years, in which 2.2% had high-grade CIN. However, the difference was not significant compared to the results of the control group^17^. Owens et al. followed up 1,442 cases of unsatisfactory Pap smears. Among them, 770 patients underwent follow-up testing within 120 days. The risk of CIN2 + was similar in patients with both unsatisfactory and satisfactory Pap test results. However, a positive HPV test was found to be the strongest risk factor for developing CIN2 + -related disease. In contrast, a negative HPV test result was protective for a CIN2 + diagnosis^18^. López-Alegría et al. followed up 1285 women who had previous unsatisfactory Pap smear results for 1 year and concluded that it was not necessary to repeat the Pap test early on, except for in cases with unsatisfactory results due to hemorrhagic and inflammatory cytological obscureness^19^. In our study, all 67 2-year follow-up tests were negative for malignancy. One of the 240 cases, who previously had a normal Pap result, was positive for CIN3 + at the follow-up testing (0.4%). No differences in secondary outcomes between the normal and unsatisfactory Pap smear groups were found in our study. The results are summarized in Table 5.

**Table 5:** Previous studies of follow-up outcomes of an unsatisfactory pap smear.

Study	Population number (inadequate/total)	Follow-up time	Results
Adams et al.^16^	172/23,302	5 years	25 (14.5%) had squamous abnormalities Atypical squamous cells (22) Low-grade squamous intraepithelial lesion (2) High-grade squamous intraepithelial lesion (1) No differences in the incidence of squamous abnormalities compared to control
Hock et al.^17^	1972 (inadequate)	5 years	2.2% high grade CIN The difference was not significant with adequate cohort (1.3%)
Owens et al.^18^	770/634,644	120 days	The risk of CIN2 was similar for patients with both unsatisfactory and satisfactory Pap tests
López-Alegría et al.^19^	1285/2547	1 year	85.9% of the women had normal smears (1104/1285) 0.6% obtained cytological reports of cervical atypia (*n* = 8) 10.9% had a repeat test after an inadequate smear (*n* = 139) 1.6% had a low-grade cervical lesion (*n* = 21) 1.0% had a high-grade lesion (*n* = 13) Not necessary to repeat the Pap test early on, except for inadequate hemorrhagic and inflammatory cytological results

CIN: cervical intraepithelial neoplasia.

For cervical cancer screening, HPV testing is recommended as the primary screening tool^3^. HPV test is checked for high-risk types of HPV infection in cells^3^. HPV testing is more sensitive than cytologic tests (smear or cytology tests) in cervical cancer screening^20^. The meta-analysis showed HPV test could increase the detection rate of CIN2 or higher, which can prevent cervical cancer^21^. An extended screening interval is also suggested due to the early detection of precancer lesions by HPV testing. An observational study of English screening pilot data also showed extension of cervical screening interval with primary HPV testing was feasible^20^. For women aged from 25 to 65 years, cervical cancer screening can be done with HPV testing every 5 years. If HPV test alone is not accessible, cotesting HPV with Pap smear every 5 years or a Pap test every 3 years are suggested by American Cancer Society^22^. After proper sampling, HPV test used as primary screening test can also decrease the chance of scant cellularity.

There are some limitations. Only 315 women were included in our study after excluding patients who did not have enough demographic information or were not available for a follow-up 2 years later. Second, Pap smears were collected from procedures performed by eight obstetricians/gynecologists and eleven PGY physicians in one medical center, and some of them might be unfamiliar with the sampling technique which could have resulted in unsatisfactory results. The high loss follow-up rate in the unsatisfactory Pap group was noted. We speculated the high loss follow-up rate might be the women did not recognize the potential risk of unsatisfactory Pap. However, based on the previous studies and our study, the risk of precancer and cancer after 2 years is little.

## Conclusions

Unsatisfactory Pap smear results might be influenced by pre-menopause status and increased vaginal discharge. Clinicians must be aware of these risks and encourage using liquid-based cytology techniques (eg: SurePath or ThinPrep) to improve scant cellularity^23^. Proper sampling and the use of HPV testing as a primary screening test was also recommended. The results of our study revealed that the Pap smear’s unsatisfactory results due to scant cellularity did not increase the risk of intraepithelial neoplasia or cancer, after 2 years. However, our study population was too small to deduce the risk of cancer risk in women with unsatisfactory Pap results. Larger prospective cohort studies with longer follow-up duration should be conducted.

## Data Availability

All relevant data were shown in the manuscript.
